# Supplementary material for: Malaria and dengue fever in febrile children entering healthcare facilities in Mwanza, Tanzania
Source: PLoS One. 2024 Oct 11;19(10):e0309613. doi: 10.1371/journal.pone.0309613 (PMC11469492; doi:10.1371/journal.pone.0309613)
Supplement: Attachment — Submitted filename: Response to Editor comment.docx [file pone.0309613.s002.docx]

**RESPONSE TO THE EDITORS’ COMMENTS FOR** **PONE-D-24-14812**

Title: Malaria and dengue fever in febrile children entering healthcare facilities in Mwanza, Tanzania

PLOS ONE

Response to the Editors’ comment

We thank the Editors for their helpful comment. Find our response to this comment below.

**Editor comment**

1. At this time, please confirm whether your submission contains your "minimal data set", which PLOS defines as consisting of the data set used to reach the conclusions drawn in the manuscript with related metadata and methods, and any additional data required to replicate the reported study findings in their entirety. This includes:

a) The values behind the means, standard deviations and other measures reported;

b) The values used to build graphs;

c) The points extracted from images for analysis.

- ***We confirm that the data submitted in excel form comprise the minimum data set. The do-file for Stata data analysis output and all files were uploaded in the Supplementary File.***
